# Professionals’ views of fetal monitoring during labour: a systematic review and thematic analysis

**DOI:** 10.1186/1471-2393-12-166

**Published:** 2012-12-27

**Authors:** Valerie Smith, Cecily M Begley, Mike Clarke, Declan Devane

**Affiliations:** 1 School of Nursing & Midwifery, University of Dublin, Trinity College Dublin, 24, D’Olier St, Dublin, Ireland; 2 All-Ireland Hub for Trials Methodology Research, Centre for Public Health, Queen’s University Belfast, Northern, Ireland; 3 School of Nursing & Midwifery, National University of Ireland, Galway, Ireland

**Keywords:** Fetal monitoring, Pregnancy, Labour, Views

## Abstract

**Background:**

Current recommendations do not support the use of continuous electronic fetal monitoring (EFM) for low risk women during labour, yet EFM remains widespread in clinical practice. Consideration of the views, perspectives and experiences of individuals directly concerned with EFM application may be beneficial for identifying barriers to and facilitators for implementing evidence-based maternity care. The aim of this paper is to offer insight and understanding, through systematic review and thematic analysis, of research into professionals’ views on fetal heart rate monitoring during labour.

**Methods:**

Any study whose aim was to explore professional views of fetal monitoring during labour was considered eligible for inclusion. The electronic databases of MEDLINE (1966–2010), CINAHL (1980–2010), EMBASE (1974–2010) and Maternity and Infant Care: MIDIRS (1971–2010) were searched in January 2010 and an updated search was performed in March 2012. Quality appraisal of each included study was performed. Data extraction tables were developed to collect data. Data synthesis was by thematic analysis.

**Results:**

Eleven studies, including 1,194 participants, were identified and included in this review. Four themes emerged from the data: 1) reassurance, 2) technology, 3) communication/education and 4) midwife by proxy.

**Conclusion:**

This systematic review and thematic analysis offers insight into some of the views of professionals on fetal monitoring during labour. It provides evidence for the continuing use of EFM when caring for low-risk women, contrary to current research evidence. Further research to ascertain how some of these views might be addressed to ensure the provision of evidence-based care for women and their babies is recommended.

## Background

Current research demonstrates a lack of evidence of benefit for the use of electronic fetal monitoring (EFM) compared to intermittent auscultation (IA) of the fetal heart rate (FHR) during labour [1,2]. Despite this, EFM remains widespread in clinical practice [3,4]. In considering barriers to and facilitators for implementing evidence-based healthcare, the thoughts, views, perspectives and experiences of individuals concerned directly with those interventions are important. This is because exploring individual perspectives can offer insight and understanding that might not be captured by experimental research, which focuses primarily on clinical outcomes. Furthermore, it may offer some explanations, from a user’s perspective, on the use and choice of FHR monitoring modalities in practice, especially when this is contrary to current recommendations. Evidence-based practice is not merely about the application of research evidence, but needs to incorporate values, preferences and experiences of both the practitioner and the person being offered care. In this sense, consideration can be given to the findings of research inclusive of reasons for not adopting the findings within clinical practice. For these reasons, this paper offers a systematic review and thematic analysis of research into professionals’ views of fetal monitoring during labour. A synthesis of women’s views of FHR monitoring during labour is reported separately.

### Aim

To offer insight and understanding, through summary, aggregation and interpretation of findings from studies that report on professionals’ views, experiences and/or perspectives, on FHR monitoring during labour (where professional refers to midwife, obstetrician and/or obstetric nurse).

## Methods

Systematic review is a research method that compares individual research studies on similar topics and summarises their findings in a single report. They are often limited to reports of quantitative studies, such as randomised trials, with pooling and statistical analysis (meta-analysis) of study results [5]. Meta-synthesis of findings from qualitative enquiry is gaining momentum and examples of such synthesis are apparent in the literature [6-8]. One benefit of considering the findings from synthesis of qualitative enquiry, in conjunction with the findings from synthesis of quantitative research, is the potential for the increased implementation of evidence-based practice and the implications this may have for future care [5,9,10].

A framework, used previously by the Evidence for Policy and Practice Information and Coordinating (EPPI) Centre at the Institute of Education in London, in their synthesis of children’s views on healthy eating [11], was used to guide our systematic review of professionals’ views of fetal monitoring during labour.

### Search and selection strategy

**Figure 1:**
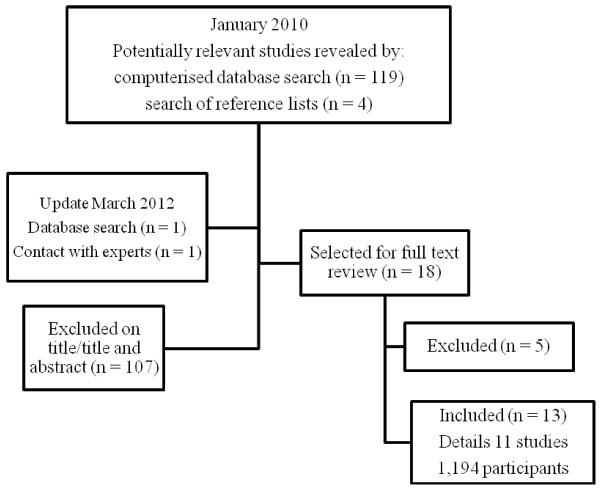
Search and Selection Strategy.

The electronic databases of MEDLINE (1966–2010), CINAHL (1980–2010), EMBASE (1974–2010) and Maternity and Infant Care: MIDIRS (1971–2010) were searched in January 2010 and in March 2012 (updated search), using the keywords; ‘*fetal monitoring*’, ‘*labour*’, ‘*pregnancy*’ ‘*perceptions*’, and ‘*views*’. These were combined using the Boolean operand ‘AND’ (for example, ‘fetal monitoring’ AND ‘views’ AND ‘pregnancy’). Papers were then discarded, or selected for full text review, based on their relevance as judged by title, or title and abstract. Reference lists of all full text papers were studied to identify potentially relevant studies not captured by the electronic search [12]. Only English language publications were retrieved, due to a lack of access to language translators at the time of conducting the review. Figure 1 outlines the search and selection strategy.

### Inclusion/exclusion criteria

To overcome the potential problems associated with methodological incomparability of ‘views’ studies, where different methods might have been used to explore the phenomenon of interest, our inclusion criterion was based on the aim of the review rather than on study design or method [13]. In this sense, all studies, regardless of study design, whose aim was to explore and report on professionals’ views or experiences of, or attitudes towards, any method of FHR monitoring in labour were eligible. A total of 11 studies were identified and included. The characteristics of these studies are described in Tables 1 and 2.

**Table 1:** Summary characteristics of included studies

**Author/Year**	**Aim**	**Participants and study location**	**Key findings reported by authors**	**Key themes identified by reviewer**
Cranston, 1980 [29]	To identify the attitudes of professionals towards fetal monitoring	124 obstetric nurses; 14 hospitals, St Louis, USA	88% felt that **fetal surveillance** by the FM could not be achieved by IA. 90% felt that the woman was **more reassured by the presence** of the monitor. 59% did not feel that the FM causes more **patient anxiety**. 98% felt that the purpose of the FM was **to improve fetal outcome**. EFM is one of obstetrics **best inventions**.	Reassurance
				Safety
				Technology
				Anxiety
Dover and Gauge, 1995 [14]	To find out how midwives carried out intrapartum FHR monitoring and what factors influenced choice of methods	117 midwives of 242 (48% response rate); 3 units, England	Midwives felt **confident to use IA** for low-risk women; midwives would **benefit from education on EFM interpretation**; philosophy of childbirth affected choice of method; EFM was used when **staffing levels were poor**.	Reassurance
				Education
				Monitor as midwife
Birch and Thompson, 1997 [28]	To determine staff attitudes to and practice of monitoring the FHR during labour	96 professionals (14 doctors, 80 midwives, 2 unknown), (50% response rate); Consultant led unit, Wirral, England	EFM has **improved outcomes**; overall preference for IA; disparity between midwives’ and doctors’ responses.	Reassurance
				Safety
Sinclair, 2001 [32]	To explore how midwives used the birth technology of the CTG machine	446 midwives of 741 (60% response rate); All labour wards, Northern Ireland	Dichotomy with respect to reliance on EFM and EFM as a source of **anxiety**; view that CTG is not required for **safe birth**; agreement that **technology in childbirth** is desirable.	Reassurance
				Anxiety
				Technology
Walker et al, 2001 [25]	To explore nurses’ attitudes towards IA	145 obstetric nurses; 5 units, South-East Michigan, USA	IA should be the standard of care; **staff**/**women ratios hinder IA** use; neutral response to **research on EFM** and clear benefits.	Education
				Monitor as midwife
Munro et al, 2002 [24]	To explore and respond to midwives’ views of different types of fetal monitoring in labour	20 midwives; 2 maternity units, England	EFM offered **reassurance**; **increased anxiety**; EFM can hinder **communication**; EFM **reduces mobility and increased need for pain relief**; **trust in technology**.	Reassurance
				Anxiety
				Communication
				Technology
Altaf et al, 2006 [31]	To explore midwives’ views on the experience of using EFM	20 midwives; large teaching hospital, England	Feeling of **reliance on EFM**; EFM can erode and undermine professional skills; EFM **deflecting attention from care**.	Reassurance
				Technology
				Communication
Hindley et al, 2006* [20]	To explore midwives’ attitudes and experiences of intrapartum fetal monitoring	58 midwives; 2 hospitals, northern England	Midwives were motivated to use EFM to **protect themselves against** potential **litigation**; EFM may provide **reassurance**; IA allowed for **closeness to women** and **freedom of movement** during labour; IA facilitated a more natural approach to childbirth; danger of losing skills with **over**-**reliance on technology**; EFM used when **busy**.	Reassurance
				Litigation
				Communication
				Technology
				Monitor as midwife
Blix and Ohlund, 2007 [26]	To explore what information the labour admission test is perceived to provide in the daily work of midwives	12 midwives; four maternity units, Norway	The core category ‘experiencing contradictions’ was explained by three sub-categories; professional identity **versus technology**, feeling **safe versus feeling unsafe** and power versus powerlessness.	Safety
				Technology
McKevitt et al, 2011 [30]	To examine midwives’ and doctors’ attitudes towards the use of the CTG machine in labour ward practice	29 of 56 midwives (52%) and 11 of 19 doctors (58%) (survey); 6 midwives and 2 doctors (interviews); maternity unit, Northern Ireland	Questionnaires: CTGs lead to unnecessary interventions; disagreement that **technology in childbirth is undesirable**; agreement re use of CTG not **distracting attention from mother**; CTGs used unnecessarily; disagreement in always **trusting the CTG and in feeling vulnerable** without it; **decision**-**making** for intervention; Interviews: determining appropriate usage-CTG monitoring used to **provide reassurance**; reaching a decision, **communication and collaboration on CTG interpretation**; professional concerns-limited evidence to support CTG use, increased intervention; the way forward-**more research to improve technology to monitor the fetus**.	Technology
				Communication
				Reassurance
				Education
Hill, 2011 [27]	To explore midwives’ views and experiences of using intermittent auscultation of the fetal heart during labour	8 midwives; large urban maternity unit, Ireland	Lack of **policies and guidelines** on use of IA; need to **provide proof of the FHR**; **vulnerable to litigation**; culture of the organisation; medicalisation, **industrialised birth and technology**; walking a tightrope = dilemma of wanting to use IA, **busy clinical environment** and **feeling vulnerable**	Communication
				Reassurance
				Litigation
				Technology
				Monitor as Midwife

*The results of this study are reported across three publications; references for additional papers include Hindley and Thomson [21] and Hindley & Thompson [22].

**Table 2:** Methodological characteristics of included studies

**Author/Year**	**Sampling methods**	**Data collection**	**Data analysis**
Cranston, 1980 [29]	Non-probability	Questionnaire (24-item Likert scale)	One-way ANOVA, mean, standard deviations and frequency counts
Dover and Gauge, 1995 [14]	Non-probability	Questionnaire (20-item Likert scale)	ANOVA, frequencies, correlation, chi-square and t-tests
Birch and Thompson, 1997 [28]	Non-probability	Questionnaire (in-hospital survey)	Frequencies
Sinclair, 2001 [32]	Non-probability	Questionnaire (postal survey, 25-item Likert scale)	Descriptive, Factor analyses
Walker et al, 2001 [25]	Non-probability	Questionnaire (18-item Likert scale)	ANOVA, mean, standard deviation
Munro et al, 2002 [24]	Non-probability	Semi-structured interviews	Framework analysis
Altaf et al, 2006 [31]	Non-probability	Semi-structured interviews	Constant Comparative Method
Hindley et al, 2006* [20]	Non-probability	Semi-structured interviews	General thematic analysis
Blix and Ohlund, 2007 [26]	Non-probability	Interviews	Constant Comparative Method
McKevitt et al, 2011 [30]	Non-probability	Questionnaire (postal survey, 25-item Likert Scale) and Interviews	Frequencies and thematic analysis
Hill 2011 [27]	Non-probability	Semi-structured interviews	Colaizzi’s Methods with themes formulated

### Quality assessment

Guided by the framework offered by the EPPI-Centre, we performed a quality assessment of each included study in this review, using the EPPI-Centre’s 12 assessment criteria (Table 3).

**Table 3:** Quality assessment of included studies

**Study**	**Quality criteria met**
Cranston, 1980 [29]	A, B, C, F, H, J, K
Dover and Gauge, 1995 [14]	A, B, C, D, E, F, G, H, I, J, K, L
Birch and Thompson, 1997 [28]	A, C, D, J,
Sinclair, 2001 [32]	A, B, C, D, E, F, G, H, I, J, K, L
Walker et al, 2001 [25]	A, B, C, D, E, F, G, H, I, J, K
Munro et al, 2002 [24]	A, B, C, D, E, F, G, H, I, J, K, L
Altaf et al, 2006 [31]	A, B, C, D, E, F, H, J, K
Hindley et al, 2006 [20]	A, B, C, D, E, F, G, H, I, J, K
Blix and Ohlund, 2007 [26]	A, B, C, D, E, F, G, H, I, J, K
McKevitt et al, 2011 [30]	A, B, C, D, E, F, J, K
Hill, 2011 [27]	A, B, C, D, E, F, G, H, I, J, K

***Quality of study reporting***.

A: Aims and objectives were clearly reported.

B: Adequate description of context of research.

C: Adequate description of the sample and sampling methods.

D: Adequate description of data collection methods.

E: Adequate description of data analysis methods.

***There was good or some attempt to establish the***:

F: Reliability of data collection tools.

G: Validity of data collection tools.

H: Reliability of data analysis.

I: Validity of data analysis.

***Quality of methods***.

J: Used appropriate data collection methods to allow for expression of views.

K: Used appropriate methods for ensuring the analysis was grounded in the views.

L: Actively involved participants in the design and conduct of the study.

### Data extraction

Data extraction was based on the review question; that is, professionals’ views of FHR monitoring during labour. Criteria for data to be extracted (Table 1 and Table 2) were predetermined. These tables facilitated the presentation of each study in a standard format and enabled comparisons between studies and summary aspects of the review. The data extraction process involved a careful line-by-line review, complete immersion in, and breakdown of findings for each included study. This process was time-consuming, but essential, as the reports of included studies varied in style and format. By carefully deconstructing the findings of each study, we were able to identify and retrieve relevant data meeting the review’s aim.

### Data analysis

Thematic analysis of each study’s findings was carried out. Identification of prominent or recurrent themes in the individual studies was followed by an amalgamation and synthesis of the findings under thematic headings. Thematic analysis has been praised for allowing considerable latitude to reviewers, and for enabling the integration of findings from qualitative and quantitative enquiry [14]. Considering the potential for including studies of both quantitative and qualitative design, we deemed thematic analysis to be the most appropriate form of analysis to meet the aim of our review.

The steps used to conduct thematic analysis were adopted from Lucas et al [7] as follows;

1. We extracted data from the included studies’ findings and entered them into a table (Table 1).

2. We then reviewed the data in Table 1 and isolated emergent themes from each study’s findings.

3. A list of themes was documented for each study (Table 1, last column). To clarify the association between findings and themes, the relevant section of findings was highlighted in bold.

4. A synthesis of all findings was then performed.

To ensure accuracy and reliability in reporting the findings, we engaged in an iterative process of data synthesis, which involved going back and forth between the data extraction tables and the original articles.

## Results

### Search & selection strategy

The search strategy identified 125 articles. Of these, 107 were excluded on the basis of their title or abstract because they clearly did not identify professionals’ views of FHR monitoring during labour. Eighteen citations remained for full text retrieval and review. Following this, a further 5 were excluded; one explored midwives’ perceptions of the use of technology and was not explicitly about monitoring the FHR [15], one was a duplicate publication [16], one explored views on decision-making and was not explicitly about monitoring the FHR [17] and two were either non-comparable in design or it was impossible to extract themes from the data [18,19]. In total, 13 papers, detailing 11 studies (1,194 professionals), are included in our review (Figure 1); the findings of one study [20] are reported across two additional publications [21,22]. Details of the studies included are outlined in Table 1 and Table 2.

### Quality assessment

Table 3 presents the results of the quality assessment of included studies. Three studies, [15,23,24] reported all 12 of the EPPI-Centre’s quality assessment criteria in their papers, indicating ‘good’ quality studies. Four studies, [20,25-27] addressed 11 of the 12 criteria in their papers. The study by Birch and Thompson [28] scored the lowest on quality assessment, addressing only four of the 12 criteria. The remaining studies addressed seven [29], eight [30] and nine [31] of the 12 quality assessment criteria.

### Professionals’ views of FHR monitoring

Thematic analysis and synthesis of each included study resulted in the emergence of four prominent themes related to professionals’ views on fetal monitoring during labour. These were: 1) reassurance and safety, 2) technology, 3) communication/education and 4) midwife by proxy.

#### Reassurance and safety

Reassurance emerged as a prominent theme in 9 [15,22-24,27-31] of the 11 included studies. EFM offered reassurance because professionals believed the cardiotocograph (CTG) trace provided hard copy ‘proof’ that the baby was not compromised whilst in their care [22-24,26,27,31]. When an adverse outcome occurred, this ‘proof’, not achievable through IA, was perceived as potentially minimising the exposure of clinical staff to criticism and litigation [22,26,27]. From the data, it appeared that a principal reason for using EFM was the perceived reassurance (‘*the ability to hear the fetal heart in the background*’ [30]) and perceived protection against legal action afforded by the hard copy CTG trace [21-23,26,27,31];

‘*The main disadvantage I can see for using intermittent auscultation is from a litigation point of view*; *it*’*s your word against theirs if there*’*s a problem because you*’*ve not got the proof*…*you haven*’*t got the CTG to look at*’. [22], (p.236)

‘…*there is a kind of fear in some ways about if*……*something happens and they say* “*well where is the record*, *where is the proof*”’ [27], (p.39)

‘…*it gives you a nice hard copy and I think that suits everybody*, *it just settles your mind and you*’*ve got proof and with intermittent you have no copy*’ [31], (p.414)

Professionals’ faith in the safety of EFM in assuring improved outcomes is also evident in some of the included studies. For example, in Cranston’s [29] study, 47% strongly agreed and 41% agreed that ‘*the fetal surveillance achieved by fetal monitoring cannot be matched by intermittent auscultation*’ (p.346). In Birch and Thompson’s [28] study ‘*on the whole respondents felt that the use of EFM improved outcome for all women*…’(p.734). In a further two studies, 96% [28] and 63% [18] of professionals believed that EFM reduced perinatal mortality and morbidity and improved maternal and neonatal outcomes.

In contrast, however, a proportion of professionals in other studies believed that EFM did not necessarily ensure a good neonatal outcome. In Dover and Gauge’s [23] study, for example, professionals who considered childbirth as a normal life event did not agree that continuous EFM was safer than IA (r = 0.3107, p = 0.001). In Sinclair’s [15] study, 80% (n = 357 of 446) of professionals disagreed that a CTG was required for a safe birth. McKevitt’s study [30] reported similar findings, with 90% of professionals (n = 36 of 40) disagreeing that CTGs are ‘*essential for successful deliveries*’ and 67.5% (n = 27 of 40) disagreeing that they felt vulnerable if a CTG was unavailable. In addition, despite being reassured by the visual aspect of the CTG [24], professionals also believed that EFM can provide a false sense of security [26] with the majority of professionals in the Sinclair [15] and McKevitt [30] studies indicating that they would not always trust the CTG trace over their own observations (85% and 80% respectively).

#### Technology

Technology as a theme emerged in 8 [15,20,25-27,29-31] of the 11 included studies. EFM was considered by some as a technology offering more authoritative information than IA, but this was rejected by others. In Cranston’s study [29], for example, 80% of the 124 professionals felt that fetal assessment achieved by EFM cannot be matched by IA and 76% believed that the fetal monitor was one of obstetrics’ best inventions. In contrast, 85% of the 446 professionals in Sinclair’s study [15] disagreed that they would always trust the CTG over their own observations, and 74% felt that the CTG was often used unnecessarily. Similarly, in McKevitt’s study [30], 70% of professionals felt that the CTG was often used unnecessarily and 82.5% agreed that CTGs can lead to unnecessary medical intervention. Increased routine intervention in childbirth and the increased use of additional birth technology (e.g. epidurals, use of oxytocin and centralised monitoring) also emerged as influential factors in determining FHR monitoring modality during labour;

‘*our epidural rate is high enough too*, *so all lends to the using of continuous monitoring*’ [27], (p.43)

Concern was additionally expressed from professionals that EFM technology and over-reliance on the CTG was eroding traditional midwifery skills such as use of the Pinard fetal stethoscope [26,31,32] and this was seen as detracting from normality in childbirth [20];

‘*No*. *I*’*m not as skilled as I used to be because technology has taken over so much*. *But I try*, *to keep my skills*, *in using the Pinard*’ [26] (p.52)

‘*I think IA brings you closer to them*, *and it*’*s just more natural and normal*, *so it*’*s less technology that I am in favour of*’ [20], (p.357)

In addition, professionals also shared the view that EFM technology was more restrictive and uncomfortable than IA [20,24,31] and that it leads to increased requests for pain relief [20,24];

‘*I think especially with monitors*, *they are waiting for the next pain*. *The focus is on the pain*’ [20], (p.357).

#### Communication/Education

Professionals expressed concern that EFM technology can become the focus of care and that this might distract from the care provided to women and hinder effective communication with women [20,25,26,31].

‘*It takes your attention away from the woman because you*’*re anxious that you need to keep looking at it*…’ [31], (p.416)

‘*Everybody in the room focuses on it*, *if the conversation dries up everybody looks at the monitor*’ [31], (p.416)

Professionals expressed a preference for using IA because it facilitated freedom of movement [20] and increased the experience of ‘closeness’ with women [27] and reduced anxiety associated with EFM;

‘*I suppose if you*’*re listening in intermittently*…*you*’*re adopting to their position*. *It*’*s probably a bit more like hands on touch maybe and involvement*….*you probably feel closer to them because there*’*s that extra little touch element there*’ [27], (p.44)

‘*You*’*re listening in every 15 minutes so you*’*re gonna have that communication with her and talk to her*…’ [27], (p.44)

In McKevitt’s study [30], 60% of professionals believed that using the CTG increased women’s anxiety levels; however, in another study [32], professionals were divided equally (40% agreed and 40% disagreed) on this point.

However, the use of routine interventions in childbirth and the culture of the organisation hindered the effective implementation of this practice;

‘*If* [doctors] *haven*’*t got something to look at they don*’*t want to know*’ [31], (p.416)

Evidence concerning educational issues associated with fetal monitoring modalities and their use in practice emerged from 4 [23,25,27,30] of the 11 included studies. Participants in Dover and Gauge’s study [23], for example, indicated that professionals would benefit from education on EFM interpretation, while professionals interviewed in McKevitt’s study [30] felt that communication and collaboration on CTG interpretation was required for clinical decision-making. A lack of guidelines and policies on IA use was also highlighted in one study [27], while professionals in other studies indicated that more research was required for improving fetal monitoring [30] and for identifying clear benefits for EFM use [25].

#### Midwife by proxy

Using EFM as a substitute for midwifery staff emerged from 5 of the 11 included studies [20,23-25,27] and was associated strongly with poor staffing levels and busy clinical environments;

‘*It can be used so you can go out and look after your fourth patient and come back in and see that the baby has been alright at the time you have gone*…’ [24], (p.497)

‘*It*’*s busy*, *it*’*s sometimes easier to have them on the monitor*, *epiduralised*, *at least you know what*’*s going on if you*’*re running inbetween rooms*’ [27], (p.44)

‘….*I just took that trace off and then she was like* “*oh*……*what if there*’*s something going on and you don*’*t get a chance to go in you better leave the monitor on so I can watch it from here*”’ [27], (p.45)

This sentiment is quantified in the Dover and Gauge [23] and Walker [25] studies where 72% (n = 84) and 54% (n = 78) of professionals, respectively, felt that EFM was more likely to be chosen when midwife-to-women ratios were reduced.

## Discussion

This systematic review and thematic analysis has identified themes related to professionals’ views of monitoring the FHR during labour through a synthesis of 11 studies on this topic. Four prominent themes (reassurance and safety, technology, communication/education and midwife by proxy), which might be considered influential when attempting to implement evidence-based FHR monitoring practices during labour, emerged from the data.

EFM offered professionals reassurance because they perceived it as providing the hard copy ‘proof’ of an uncompromised baby. This ‘proof’ was perceived to minimise exposure to criticism and potential litigation. However, professionals also recognised the false sense of security offered by EFM and not all professionals relied on the CTG to ensure a good neonatal outcome. The view that EFM offered reassurance of an uncompromised baby appeared to change over time. The earlier studies, for example, Cranston [29], and Birch and Thompson [28], demonstrated more faith, by professionals, in EFM assuring a good outcome than later studies [26,30,32]. This may reflect the lack of evidence of benefit on the safety and efficacy of EFM over IA that has emerged from randomised trials [1] during this period of time. In addition, evidence reporting variations in inter- and intra-observer agreement in CTG interpretation has emerged [33,34] since the publication of the earlier studies and this may have affected some professionals’ confidence in EFM use.

Determining choice for or differentiating between types of FHR surveillance based on perceptions of risk as influenced by feelings of safety and reassurance can pose challenges for professionals in clinical practice. This is because the notion of risk in maternity care remains ill-defined and ambiguous and is often made more complex by professionals interpreting risk in very different ways depending on knowledge and past experiences [35]. The identified need by professionals, to have hard-copy proof of FHR surveillance, as a perceived safety mechanism and as a potential protector against possible litigation, might be overcome by recent developments in FHR monitoring technology. These developments include the ability of hand-held Doppler devices to sequentially store information on FHR auscultations and in some instances produce paper print-outs of intermittent FHR recordings. This ‘paper-proof’ could potentially facilitate the choice of IA over EFM as it addresses the concern, to some extent, of safety and reassurance when performing FHR monitoring in clinical practice.

Professionals reported a preference for IA, yet also reported difficulty in using IA due to poor staffing levels, busy clinical environments and the increased medicalisation or industrialisation of childbirth. Contrary to this, and although we have found no evidence in the literature supporting this view, it might be plausible to assume that EFM itself is increasing professionals’ time requirements and requires more time than when using IA in practice. For example, the time taken to maintain EFM equipment, respond to alarms and interpret the CTG trace, could, in practice, take much longer than the time required to record the FHR by IA. Furthermore, if EFM causes increased discomfort leading to an increased need for regional analgesia, then this will require increased observation by clinical staff and ultimately a greater commitment of time by professionals. The perceived benefits of using EFM when staffing levels are low or when the clinical environment is busy should not, however, supersede best practice guidance which recommends the use of IA for low risk women during labour [36]. In addition, professionals describe using EFM as a protector against potential litigation and as a midwife by proxy. However, applying a CTG because a professional cannot be with a labouring woman implies that a professional cannot watch the monitor, therefore reducing any protector effect potentially offered by EFM. In addition, IA allows for close proximity and engagement with women, a view highlighted by women as being very important [37,38]. This might allow for increased communication and afford professionals a greater view of the overall clinical picture. As Shearer [39] states;

‘*intrapartum fetal death is not prevented by monitors*; *it is prevented by an alert doctor* [midwife] *at the bedside of a laboring woman*’ (p.127)

Of interest in this review is the finding, in two of the included studies [30,32], of a strong agreement that CTGs are used unnecessarily (74% and 70% in these two studies, respectively) and that they can lead to unnecessary routine intervention (61% and 82.5%, respectively), compared to, in the same two studies, a reported strong disagreement that using any technology in childbirth is undesirable (75% and 82.5%, respectively). This could be interpreted as professionals experiencing conflicting attitudes, demonstrating, potentially, that consideration of the use of EFM in practice is no longer viewed as a form of intervention in childbirth, rather as a routine aspect of modern, ‘normal’ maternity care.

This review highlights some of the barriers to and facilitators for the use of IA and EFM during labour, and offers some insight and understanding of professionals’ views. These will be useful for clinical decision-makers to consider or target when implementing policy and practice change. The need to educate professionals on the most appropriate, evidence-based means of FHR monitoring for individual women, ensuring availability of FHR monitoring guidelines and policies for staff and by using a collaborative approach to fetal monitoring and CTG interpretation to ensure best practice, have been highlighted in this review. This may be assisted by highlighting that EFM has not, to date, offered any increased evidence of benefit for improved maternal and neonatal outcomes over IA for low risk women [1,2].

The availability of regular study days on fetal monitoring for all staff would provide an opportunity to discuss some of the barriers, as identified in this review, (for example, protection against and fear of litigation, poor staffing levels and busy clinical environments, increased resource requirements that can potentially result from use of EFM), to effecting evidence-based practice change.

## Conclusion

This systematic review and thematic analysis has identified themes related to professionals’ views of FHR monitoring in practice. It has offered some insight as to why EFM has such a strong foothold in professionals’ practice and in their provision of care to women during childbirth, despite the evidence that has accumulated in research studies on the comparative effects of EFM and IA. Careful consideration of professionals’ views is required as part of the process to ensure the implementation of evidence-based care and appropriate practice change in FHR monitoring during labour.

This review will be of significant benefit to policy makers, because it is the first systematic review and synthesis of evidence, that we are aware of, that brings together and considers the views, perceptions and experiences of professionals with respect to FHR monitoring during labour. It also has importance and relevance in advancing systematic review methodology, providing an additional example of the synthesis of integrated evidence from qualitative and quantitative enquiry. The views, perceptions and experiences of professionals must be considered when implementing care to effect best practice. Further research is required to establish how some of these views might be addressed to ensure that individual women receive the FHR monitoring method that is most suited to them and their needs, so that optimum care is provided to women and their infants.

## Competing interests

The authors declare that they have no competing interests.

## Authors’ contributions

All authors contributed to the rationale for and, the design of, the review. VS participated in the sequence content and drafted the manuscript. CB, MC, and DD read and critically revised the draft manuscript for important intellectual content. All authors read and approved the final manuscript.

## Pre-publication history

The pre-publication history for this paper can be accessed here:

http://www.biomedcentral.com/1471-2393/12/166/prepub
